# Biochemical and Pharmacological Characterizations of ESI-09 Based EPAC Inhibitors: Defining the ESI-09 “Therapeutic Window”

**DOI:** 10.1038/srep09344

**Published:** 2015-03-20

**Authors:** Yingmin Zhu, Haijun Chen, Stephen Boulton, Fang Mei, Na Ye, Giuseppe Melacini, Jia Zhou, Xiaodong Cheng

**Affiliations:** 1 Department of Integrative Biology and Pharmacology, Texas Therapeutics Institute, The Brown Foundation Institute of Molecular Medicine, The University of Texas Health Science Center, Houston, Texas 77030; 2 Chemical Biology Program, Department of Pharmacology and Toxicology, The University of Texas Medical Branch, Galveston, Texas 77555-0615; 3 Department of Biochemistry and Biomedical Sciences, McMaster University, Hamilton, Ontario, L8S 4M1, Canada; 4 Department of Chemistry and Chemical Biology, McMaster University, Hamilton, Ontario, L8S 4M1, Canada

## Abstract

The cAMP signaling cascade is one of the most frequently targeted pathways for the development of pharmaceutics. A plethora of recent genetic and pharmacological studies suggest that exchange proteins directly activated by cAMP (EPACs) are implicated in multiple pathologies. Selective EPAC inhibitors have been recently developed. One specific inhibitor, ESI-09, has been shown to block EPAC activity and functions, as well as to recapitulate genetic phenotypes of EPAC knockout mice when applied *in vivo*. However, a recent study raised concern that ESI-09 might act as a non-specific protein denaturant. Herein, we present a detailed biochemical and pharmacological characterization, as well as a structure-activity relationship (SAR) analysis of ESI-09. Our studies show that ESI-09 dose-dependently inhibits activity of both EPAC1 and EPAC2 with apparent IC_50_ values well below the concentrations shown to induce “protein denaturation”. Moreover, the ESI-09's action towards EPAC proteins is highly sensitive to minor modifications of the 3-chlorophenyl moiety. Taken together, these results demonstrate that ESI-09 indeed acts as an EPAC specific antagonist and does not significantly destabilize/denature proteins at pharmacological effective concentrations. This conclusion is further supported by NMR data showing that ESI-09 induces residue-dependent chemical shift changes at low concentrations, while preserving well dispersed peaks.

The cAMP binding domain (CBD) is an evolutionary conserved structural motif responsible for transducing the intracellular effects of the prototypic second messenger, cAMP. As a versatile regulatory building-block, the CBD, when coupled to different functional components, can act as a molecular switch for controlling various cellular activities^1^. The major physiological effects of cAMP in mammalian cells are mediated by two ubiquitously expressed intracellular cAMP receptor families: the classic protein kinase A/cAMP-dependent protein kinase (PKA/cAPK), and the more recently discovered exchange protein directly activated by cAMP/cAMP regulated guanine nucleotide exchange factor (EPAC/cAMP-GEF)^2^, as well as cyclic nucleotide-activated ion channels (CNG and HCN) in certain tissues^3^. Unlike PKA, EPAC proteins have no kinase activity but act as guanine nucleotide exchange factors for the down-stream small GTPases Rap1 and Rap2 in response to intracellular cAMP^4^,^5^.

The identification of EPAC proteins in 1998 ushered in a new era for cAMP-signaling research because many cAMP functions previously attributed to PKA exclusively are in fact also transduced by EPAC. Between the two mammalian EPAC isoforms, EPAC1 and EPAC2, EPAC1 is more ubiquitously expressed while EPAC2 is mainly found in CNS, pancreatic islets and adrenal gland^5^. In addition, EPAC1 and EPAC2 assume distinct cellular localizations and participate in different signalsomes^6^,^7^,^8^,^9^,^10^. As such the physiological functions of EPAC1 and EPAC2 are mostly discrete and non-redundant. A multitude of recent studies using mouse knockout models of EPAC1 and EPAC2 have revealed that EPAC proteins play important roles in energy homeostasis, cardiovascular response and pain sensing, and thus represent potential therapeutic targets for cancer, chronic pain, diabetes, and heart failure^11^,^12^,^13^,^14^,^15^,^16^.

Considering the important roles of EPAC1 and EPAC2 under both physiological and pathological conditions, developing pharmacological probes capable of selectively modulating EPAC1 and EPAC2 activity has attracted significant attentions. Through screening a library of drug-like small molecular compounds using a robust competition assay^17^, Tsalkova et al. have reported the identification of several EPAC specific inhibitors (ESIs)^18^. Among the seven ESIs identified, ESI-05 and ESI-07 are exclusively selective for EPAC2 while the remaining ESIs are pan-EPAC antagonists that inhibit both EPAC isoforms. One such inhibitor, ESI-09 has been shown to be able to selectively inhibit EPAC functions *in vitro*^19^ and *in vivo*^20^,^21^. However, a recent report has raised the concern that ESI-09 may not act on EPAC selectively^22^. In this study, we provide a detailed biochemical/pharmacological characterization and structure-activity relationship (SAR) analyses of ESI-09. Our results demonstrate that ESI-09 and its close analogs indeed act as EPAC specific antagonists by competing with cAMP binding to EPAC.

## Results

### SAR analysis

**Figure 1:**
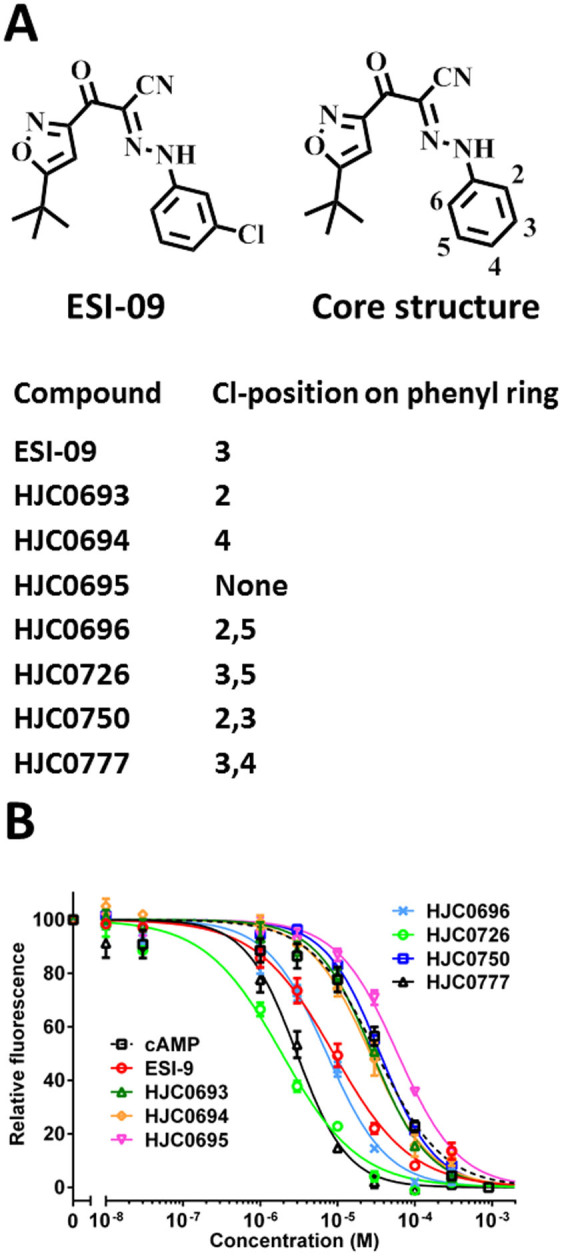
Structure-activity relationship analysis of ESI-09. (A). Chemical structure of ESI-09 and its analogs based on the core structure of 2-(5-(*tert*-butyl)isoxazol-3-yl)-2-oxo-*N*-phenyl- acetohydrazonoyl cyanide. (B) Dose-dependent competition of cAMP and ESI-09 analogs with 8-NBD-cAMP in binding to EPAC2.

ESI-09, 3-[5-(*tert*-butyl)isoxazol-3-yl]-2-[2-(3-chlorophenyl) hydrazono] -3-oxopropanenitrile, was initially identified from a high throughput screening (HTS) of the Maybridge HitFinder diversity library along with several other compounds that were able to compete with 8-NBD-cAMP (8-(2-[7-Nitro-4-benzofurazanyl] aminoethyl-thio) adenosine-3′, 5′-cyclic monophosphate) in binding to EPAC2 and specifically inhibit both EPAC2 and EPAC1's guanine nucleotide exchange factor (GEF) activity^18^,^19^. To define chemical properties important for ESI-09's specificity, we performed SAR analysis focusing on the 3-chlorophenyl moiety of ESI-09. Seven analogs with chloro-substituted at various positions of the phenyl ring were synthesized (Figure 1A), and the ability of these compounds to compete with 8-NBD-cAMP binding to EPAC2 was determined and compared to that of the parental compound ESI-09. As shown in Figure 1B, removing the 3-chloro-substituent led to a more than 6-fold decrease in potency, while 2- or 4-chloro-substituent led to a 3-fold decrease in potency, when compared to ESI-09. Furthermore, dichloro-substituent at 3- and 5-positions resulted in a 5-fold increase in potency, but dichloro-substituent at 2- and 3-positions led to a 4-fold decrease in potency (Table 1). These results suggest that 3- and 5-chloro-substituents are favorable for EPAC2 interaction, whereas 2- and 4-chloro-substituents are not.

### ESI-09 based EPAC inhibitors dose-dependently suppress full-length EPAC1 and EPAC2 GEF activity

**Figure 2:**
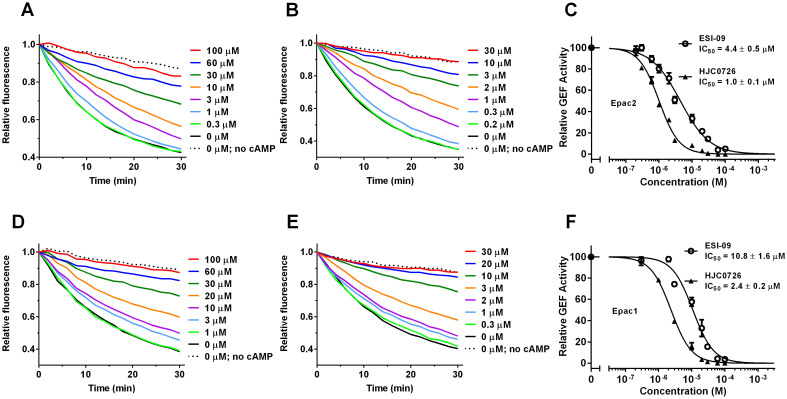
Inhibition of EPAC2 and EPAC1 GEF activities by ESI-09 and HJC0726. Dose-dependent inhibition of EPAC2 GEF activity by ESI-09 (A) or HJC0726 (B) in the presence of 20 µM cAMP. (C) Relative EPAC2 GEF activity as a function of ESI-09 and HJC0726 concentration. Dose-dependent inhibition of EPAC1 GEF activity by ESI-09 (D) or HJC0726 (E) in the presence of 20 µM cAMP. (F) Relative EPAC1 GEF activity as a function of ESI-09 and HJC0726 concentration.

The guanine nucleotide exchange activity of EPAC proteins can be biochemically determined using a coupled exchange assay, in which Rap1 loaded with a fluorescent GDP analog, 2′-/3′-O-(*N*′-methylanthraniloyl)guanosine-5′-O-diphosphate (MANT-GDP), is used as the substrate. The exchange of Rap1-bound MANT-GDP by free unlabeled GDP leads to a decrease in fluorescence as MANT-GDP contains an environment sensitive fluorophore, MANT, whose fluorescence intensity increases upon binding to Rap1. However, the MANT-GDP based GEF assay is not well suited for ESI-09 analogs because they absorb lights within the excitation emission wavelengths of the fluorophore. We recently optimized the assay replacing MANT-GDP by BODIPY-GDP, which, unlike MANT-GDP, does not interfere with ESI-09, and provides a better signal-to-noise ratio, thus allowing the assay to be performed in high throughput format using a plate-reader. As shown in Figure 2, A and C, in the presence of 20 µM of cAMP, ESI-09 dose dependently blocked cAMP-mediated EPAC2 GEF activity with an apparent IC_50_ of 4.4 µM. Furthermore, a 3,5-dichloro ESI-09 analog, HJC0726 with 5-fold increased potency in the 8-NBD-cAMP competition assay, showed similarly increased potency in inhibiting cAMP-mediated EPAC2 GEF activity with an apparent IC_50_ of 1.0 µM (Fig. 2, B & C). Similarly, ESI-09 and HJC0726 inhibited GEF activity of the full-length EPAC1 in the presence of 20 µM cAMP with apparent IC_50_ of 10.8 and 2.4 µM, respectively (Fig. 2, D–F). These results demonstrate that simple modification of ESI-09 by addition of a 5-chloro-substituent to the 3-chlorophenyl moiety, it is possible to significantly improve the binding affinity and potency of the ESI-09 compound towards both EPAC1 and EPAC2 proteins.

### ESI-09 and HJC-0726 are competitive inhibitors for full-length EPAC2

**Figure 3:**
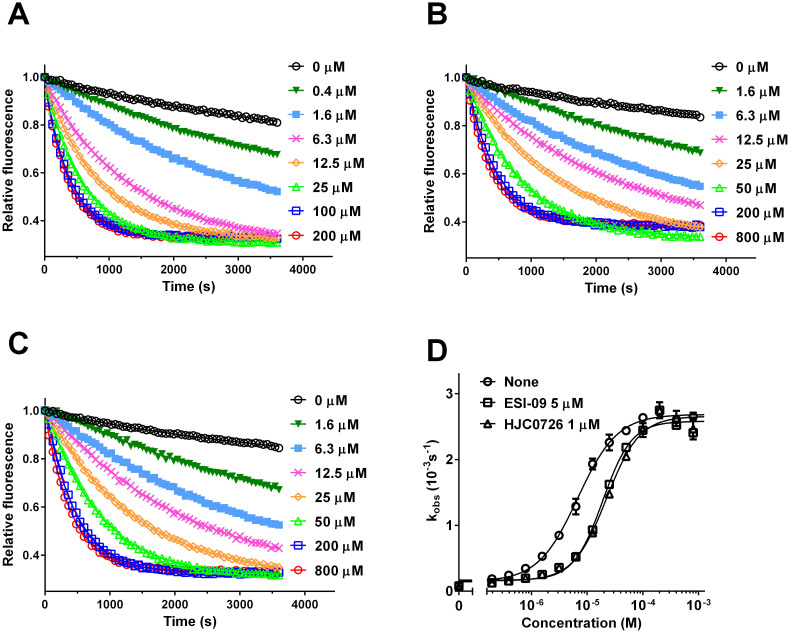
EPAC2 activation by cAMP in the absence and presence of ESI-09 or HJC0726. Dose-dependent activation of EPAC2 by cAMP in the absence of inhibitor (A), or in the presence of 5 µM ESI-09 (B) or 1 µM HJC0726 (C). (D) Relative EPAC2 GEF activity as a function of cAMP concentration.

ESI-09 was identified by a competition assay using full-length EPAC2 and 8-NBD-cAMP. To confirm that ESI-09 is indeed a competitive EPAC inhibitor against cAMP, we performed a dose-dependent cAMP titration of EPAC2-mediated Rap1 nucleotide exchange activity using the aforementioned Rap1- BODIPY-GDP assay in the presence or absence of 5 µM ESI-09. This concentration was selected on the basis of ESI-09's IC_50_ towards EPAC2. As expected, cAMP dose-dependently activated EPAC2 GEF activity with an apparent AC_50_ of 6.8 µM in the absence of ESI-09 (Fig. 3A). While high concentrations of cAMP were able to completely overcome the inhibitory effect of ESI-09 (Fig. 3B), the dose-dependent cAMP activation curve was right shifted with an apparent AC_50_ of 19.0 µM (Fig. 3D). Similar results were obtained with HJC0726 (Fig. 3C). The presence of 1 µM HJC0726 increased the apparent AC_50_ for cAMP to 22.2 µM without affecting the maximal GEF activity at saturating cAMP concentrations (Fig. 3D). These results confirmed that ESI-09 and HJC0726 acted as reversible competitive EPAC2 inhibitors toward cAMP.

### ESI-09 based EPAC inhibitors do not destabilize proteins under pharmacologically effective concentrations

**Figure 4:**
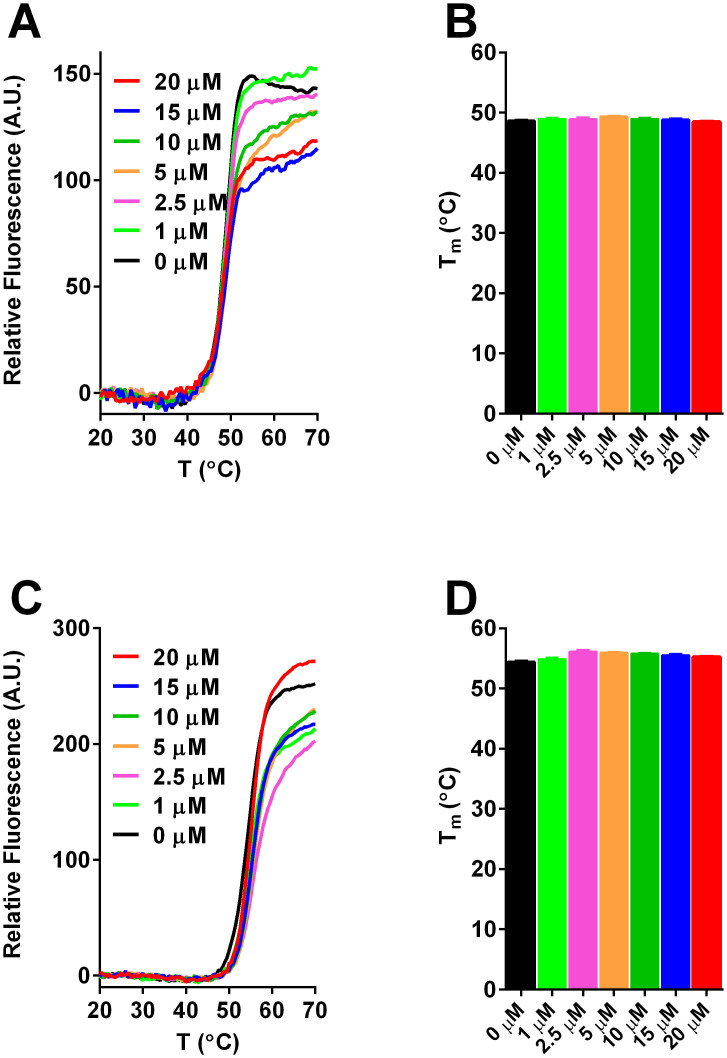
Effect of ESI-09 on thermal denaturation of EPAC2 and GST. Thermal-induced protein denaturation of EPAC2 (A) and GST (C) in the presence of various ESI-09 concentrations. Thermal melting temperature (T_m_) of EPAC2 (B) and GST (D) as a function of ESI-09 concentration.

It was proposed that ESI-09 might act also as a general nonspecific protein denaturant at high concentrations based on a protein stability analysis using a thermal-denaturation assay, in which an environmentally sensitive fluorescent dye was used to probe the thermal-induced protein unfolding^22^. It was observed that the thermal unfolding curves of various proteins up-shifted to higher fluorescent intensities when ESI-09 concentrations were kept above 25 µM. The author suggested that the higher fluorescence baselines at low temperature were the result of immediate protein denaturation upon addition of ESI-09. However, this explanation is not consistent with the fact that clear thermal unfolding transitions were still observed for all proteins examined despite the upshifts in baseline at 25 µM or higher ESI-09 concentrations. In addition, non-specific chemical denaturants, such as urea or guanidinium chloride, usually function at much higher concentrations. One more likely explanation of this elevated fluorescence baselines under high ESI-09 concentrations is the low aqueous solubility of ESI-09 as it is highly hydrophobic with a calculated cLogP of 4.54. This is a relative common property for synthetic drug leads, which usually tend to be hydrophobic in nature. For this reason, we experimentally measured the solubility of ESI-09. Indeed, the solubility limit of ESI-09 in water was determined at 5.95 µg/mL or 18 µM. Therefore, the apparent elevated fluorescence baselines observed above 25 µM of ESI-09 were most likely due to compound aggregation at high concentrations. Consequently, we performed the thermal denaturation analysis of EPAC2 and GST by keeping the ESI-09 concentrations below 25 µM. The thermo-melting curve of EPAC2 in the absence of ESI-09 exhibited a melting temperature T_m_ (defined as the mid-points of thermo-unfolding) around 48°C. Addition of ESI-09 didn't shift the T_m_ to lower temperature for EPAC2 (Fig. 4B). Similar results were observed for GST in the presence of various ESI-09 concentrations (Fig. 4C& D). We also performed the thermo-unfolding experiments in the presence of 100 µM cAMP as in Rehmann's study^22^. As shown in Figure S1, the thermo-melting curve of EPAC2 in the presence of 100 µM of cAMP shifted to left with a calculated T_m_ around 46°C. On the other hand, the presence of ESI-09 didn't lead to further changes in T_m_ (Fig. S1B). While binding of small molecule ligands usually increases the thermal stability of a protein, it is of interest to point out that binding of cAMP to recombinantly purified EPAC2 leads to a noticeable decrease in T_m_, as measured through the environmentally-sensitive dye used here. This observation suggests that cAMP interaction with EPAC causes at least a local destabilization of the protein.

**Figure 5:**
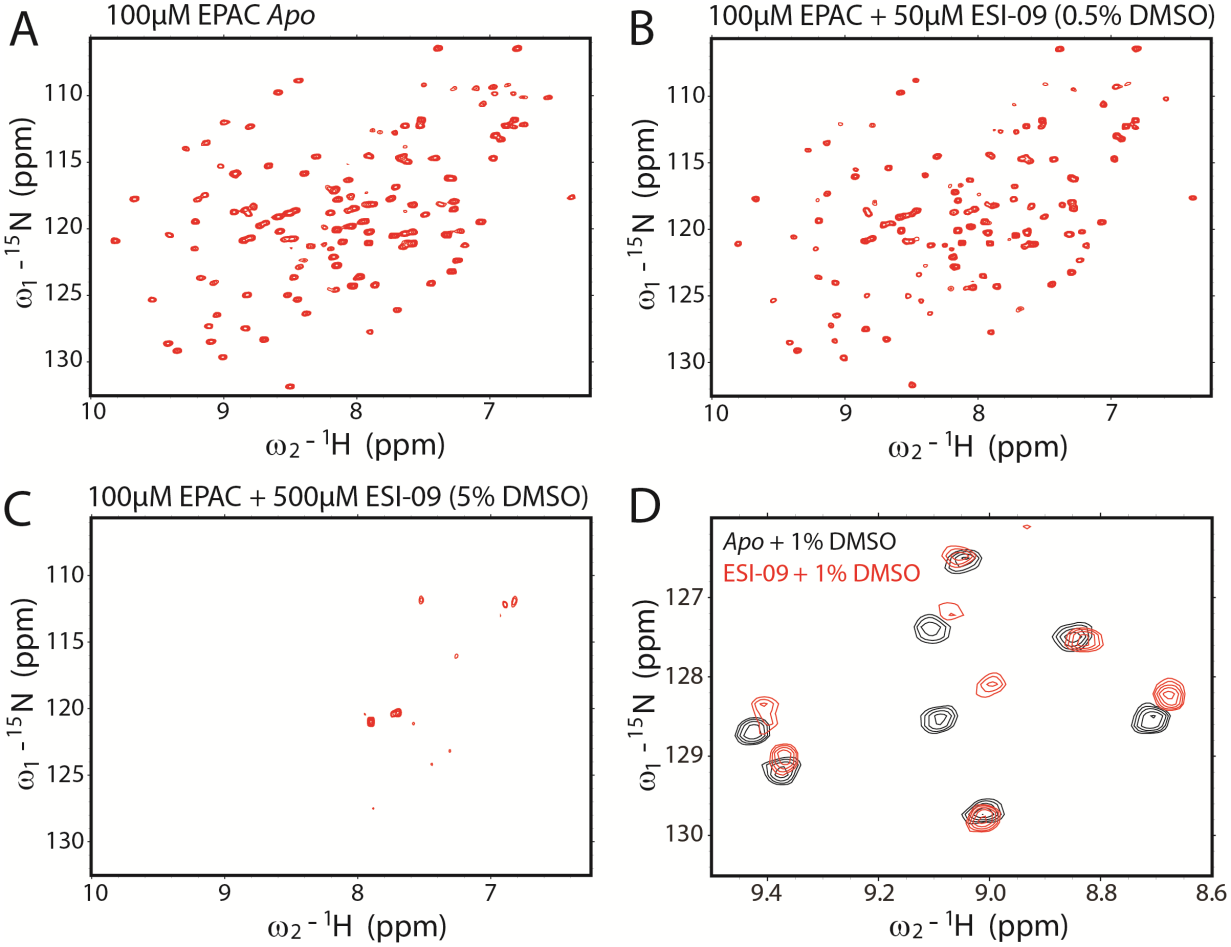
Effect of ESI-09 on EPAC1_h_ 149-318 {^15^N, ^1^H} NMR resonances. {^15^N, ^1^H}-HSQC spectra of 100 μM EPAC1_h_ 149-318 in the absence (A) and presence of 50 μM (B) and 500 μM (C) ESI-09. (D) Representative section from the spectral overlay of 25 μM *apo* EPAC (+1% DMSO) with 25 μM EPAC bound with 100 μM ESI-09 (+1% DMSO).

In addition to the thermo-unfolding experiments, {^15^N, ^1^H} Heteronuclear Single Quantum Coherence (HSQC) spectra were acquired to characterize the interaction between ESI-09 and the CBD of human EPAC1 (aa. 149-318) and address whether ESI-09 acts as a non-specific denaturant. The {^15^N, ^1^H} chemical shifts serve as excellent sensors for protein structure and dynamics as they provide information about the local chemical environment surrounding the amides of every amino acid (except prolines) within the protein. EPAC1_h_ (149-318) has already been extensively analyzed by NMR spectroscopy^23^,^24^,^25^,^26^. It results in HSQC spectra with well-dispersed peaks, which are representative of a fully folded protein (Fig. 5A). To detect what effects ESI-09 might have on the structure of the EPAC CBD, we acquired HSQC spectra of ^15^N-labeled EPAC1_h_ (149-318) in the presence of varying concentrations of unlabeled ESI-09. Only minor chemical shift changes are observed with the addition of 50 μM ESI-09 (Fig. 5B). The peaks in the spectra remain well dispersed, showing that EPAC remains folded in the presence of low concentrations of ESI-09. Intensity losses are observed for selected HSQC peaks, possibly due to intermediate chemical exchange between bound and unbound forms. However, when the ESI-09 concentration is increased to 500 μM, almost every peak broadens beyond detection. At this concentration, ESI-09 likely aggregates forming large soluble micelles, which bind the EPAC CBD and broaden its NMR signals. As a control, an HSQC spectrum of *apo* EPAC with 5% DMSO was acquired to show that the loss of signal was not due to the presence of DMSO in the sample (Fig. S2). Finally, we examined the chemical shift changes between samples whose only difference was the presence or absence of ESI-09 (Fig. 5D). There is a clear residue-dependence in the chemical shifts, indicating that there is a degree of specificity for the interaction between EPAC and ESI-09.

## Discussion

In this study, we present a thorough biochemical and pharmacological characterization of ESI-09 based EPAC specific inhibitors, provide solid evidence that ESI-09 acts as an EPAC selective antagonist by directly competing with cAMP binding, and argue against the notion that the ESI-09's effect on EPAC proteins is fully accounted for by “a non-specific protein denaturing property”^22^. Our data show that ESI-09 dose-dependently inhibits cAMP-mediated guanine nucleotide exchange activity in both EPAC1 and EPAC2 with apparent IC_50_ values well below the concentrations shown to induce “thermal unfolding shifts” reported by Rehmann^22^. Furthermore, structure-activity relationship analysis reveals that the exact position and number of the chloro-substituents on the chlorophenyl moiety are important for the potency of ESI-09 analogs in competing with 8-NBD-cAMP for EPAC2 binding. While the presence of chloro-substituent is overall favorable, modification at position 3 or 5 is more favorable than that at position 2 or 4. HJC0726 with 3, 5-dichloro-substituent is five-fold more potent than ESI-09 in inhibiting both EPAC1 and EPAC2. These results suggest that the ESI-09's action towards EPAC proteins is specific as it is highly sensitive to minor modifications of the 3-chlorophenyl moiety.

Our results further demonstrate that ESI-09 interacts specifically with EPAC proteins as a competitive inhibitor with cAMP. One major difference between our studies and Rehmann's is the cAMP concentration used in the assays. Since ESI-09 is a competitive inhibitor, its action is dependent upon ligand concentration. We used a 20 µM of cAMP, which is close to the AC_50_ of cAMP for both EPAC1 and EPAC2. On the other hand, 100 µM of cAMP, a near saturation concentration and at least one-order of magnitude higher than the physiological cAMP concentrations under stimulating conditions, was used by Rehmann. Under such high cAMP concentration, it is more difficult for ESI-09, as a competitive inhibitor, to counteract the effect of cAMP unless very high concentrations of ESI-09 are used, because ESI-09 is a competitive inhibitor that binds to the cAMP binding domain. However, ESI-09 itself has limited aqueous solubility with a maximum concentration around 18 µM (Table 2). Therefore, in aqueous media, ESI-09 will likely aggregate at a concentration higher than 20 µM (the exact solubility may be slightly affected by the DMSO content and other properties of the solution such as pH and salt concentration), which probably explain why ESI-09 appeared to act as a “general protein denaturant” at high concentrations. This conclusion was reached based on the thermal denaturation analysis performed with various proteins in the presence of 50 or 100 µM of ESI-09^22^. However, no significant changes in thermo-melting were observed by Rehmann when ESI-09 concentrations were kept under 25 µM. When we repeated the thermal denaturation analysis using EPAC2 and GST, no significant difference in thermo-denaturation could be observed when ESI-09 concentrations were kept at or under 20 µM. In fact, a slight right-shift of the mid-points of thermo-unfolding for both EPAC2 and GST at low ESI-09 concentrations. In addition, NMR experiments on the isolated CBD of EPAC1 reveal that the protein remains well-structured in the presence of ESI-09. The EPAC concentration used for these NMR experiments is significantly higher than those previously reported for the thermo-unfolding assay and may help solubilize ESI-09 *via* binding. Additionally, chemical shift changes for the ESI-09 bound state show clear residue dependence, suggesting that under our experimental conditions ESI-09 interacts with the EPAC1 CBD specifically and without denaturing it. Overall, these data suggest that under pharmacological effective concentrations, ESI-09 does not possess general protein destabilizing effects. This result is further corroborated by the preservation of the constitutive GEF activity of EPAC2 at [ESI-09] < ~ 10 µM^22^ and by the cAMP-dependent recovery of GEF activity observed here in the presence of ESI-09.

While the ESI-09 class of compounds can interact specifically with EPAC proteins at pharmacological effective concentrations, results from the current and previous studies suggest that caution should be exerted in applying these compounds in interrogating EPAC related signaling pathways. As with any pharmacological agents, therapeutic or treatment window is the key. This is particularly true for ESI-09 considering its limited aqueous solubility. We recommend making 10 to 50 mM ESI-09 stock solution using absolute DMSO, in which ESI-09 can be dissolved readily. Since ESI-09 is highly membrane permeable, for the majority of cellular applications, we suggest to keep the final ESI-09 concentration within the 1–10 µM range and not to exceed 20 µM. In addition to monitoring the desired cellular parameters, the effect of compound on cell viability should be carefully followed, and the concentration of ESI-09 should be adjusted downward if unexpected cytotoxicity was observed. A brief literature survey indicates that ESI-09 can be applied effectively to specifically suppress cellular EPAC activity in diverse biological systems under pharmacological effective concentrations well below the so-called “protein denaturation” concentrations^27^,^28^,^29^,^30^,^31^,^32^,^33^. Our recent studies also show that ESI-09 has excellent bioavailability, as well as pharmacological and toxicological profile. For instance, *in vivo* application of ESI-09 with a daily dose of 10 mg/kg IP treatment or 50 mg/kg oral gavage has been shown to recapitulate the EPAC1 knockout phenotypes of protecting mice from fatal rickettsioses^20^ and of compromising ovalbumin-induced oral tolerance^21^. Moreover, in vivo administration of ESI-09 inhibits pancreatic ductal adenocarcinoma invasion and metastasis^34^.

In summary, our results show that ESI-09 at concentrations < 20 μM acts as a specific competitive inhibitor of EPAC. Non-specific binding and potential “protein denaturation” are a concern only at higher ESI-09 concentrations. Hence, we stress the importance of keeping ESI-09 concentrations within the effective treatment window to avoid experimental artifacts at high doses due to the ESI-09's limited aqueous solubility and potential off-target effects. We are currently actively engaged in identifying potential off-targets of ESI-09 and in optimizing its pharmacological profile via medicinal chemistry efforts.

## Methods

### Reagents

8-(4-Chlorophenylthio)-2′-*O*-methyladenosine-3′, 5′-cyclic monophosphate, acetoxymethyl ester (007-AM) and 8-[[2-[(7-nitro-4-benzofurazanyl)amino]ethyl]thio] adenosine-3′,5′-cyclic monophosphate (8-NBD-cAMP) were purchased from BioLog Life Science Institute (Bremen, Germany). BODIPY-GDP (Guanosine 5′-Diphosphate, BODIPY® FL 2′-(or-3′)-O-(N-(2-Aminoethyl) Urethane), Bis (Triethylammonium) Salt) were purchased from Molecular Probes, Invitrogen (Carlsbad, CA, USA). All other reagents were purchased through Sigma-Aldrich (St. Louis, MO, USA).

### ESI-09 analogs synthesis and the solubility measurement

ESI-09 and its analogs were synthesized following the recently reported synthetic procedures^35^. The solubility of the selected compounds was measured by HPLC analysis following the reported protocol^36^.

### Protein expression and purification

Recombinant full-length human EPAC1, mouse EPAC2 and C-terminal truncated Rap1B(1-167) were purified as described previously^37^,^38^.

### 8-NBD-cAMP competition assay

The 8-NBD-cAMP competition assay was performed in 96-well microplates from Corning Costar (Cambridge, MA, USA) as described previously. Reaction mix containing 50 nM EPAC2 and 60 nM 8-NBD-cAMP in 20 mM Tris buffer, pH 7.5, with 150 mM NaCl, 1 mM EDTA and 1 mM DDT, was dispensed into 96-well plate. Test compounds at various concentrations were added to the reaction mix, and fluorescence intensity signals from the 8-NBD probe before and after the addition of test compounds was monitored using a SpectaMaxM2 microplate reader (Molecular Devices, Silicon Valley, CA, USA) with excitation/emission wavelengths set at 470/540 nm. Lastly, cAMP was added to the reaction mix at a final concentration 300 μM, fluorescence intensity was again recorded. Data were presented by nominalizing the observed fluorescence intensity (*F*) with the initial florescence signal (*F_0_*) before the addition of compound and final florescence signal after cAMP addition (*F_cAMP_*) using equation: *Relative fluorescence* = (*F – F_cAMP_)/(F_0_ – F_cAMP_) × 100*.

### In vitro guanine nucleotide exchange factor (GEF) activity assay of EPAC proteins

In vitro EPAC GEF activity was measured as previously described with the exception that purified Rap1B(1-167) was loaded with BODIPY-GDP instead of MANT-GDP^39^. The assay was performed using 500 nM Rap1b-BODIPY-GDP and 200 nM EPAC1 or EPAC2 in buffer containing 50 mM Tris-HCl pH 7.5, 50 mM NaCl, 5 mM MgCl_2_, 1 mM DTT, 50 μM GDP and the indicated concentrations of ESI-09 analogs at room temperature using half-area 96-well plates (Corning Costar 3915). The exchange reaction was monitored using a Spectramax M2 Plate Reader (Molecular Devices) with the excitation and emission wavelengths set at 485 and 515 nm, respectively. The reaction rate constant (*k_obs_*) was obtained by fitting the experimental data to a single exponential equation. The observed *k_obs_* was then plotted against cAMP concentration to determine the apparent activation constant (*AC_50_*). For EPAC inhibition studies, data were presented by nominalizing the observed *k_obs_* in the presence of inhibitor with the rate constant in the presence of 20 cAMP without inhibitor (*k_cAMP_*) and the rate constant without both cAMP and inhibitor (*k_0_*) using equation: *Relative GEF activity* = (*k_obs_ – k_0_)/(k_cAMP_ – k_0_) × 100*.

### Protein thermal denaturation assay

Thermal denaturation assay was performed using a BioRad CFX96 Touch^TM^ Real-Time PCR Detection system as described previously^22^. Briefly, 25 μl reaction mix containing EPAC2 or GST at a final protein concentration of 0.1 mg/ml, Sypro Orange at 1:1000 of the stock solution supplied by the manufacturer (Sigma) and ESI-09 at 1 μM, 2.5 μM, 5 μM, 10 μM, 15 μM or 20 μM was placed in 96 well real-time PCR plates. Fluorescence intensities with baseline subtracted were monitored over a temperature range from 11°C to 80°C with a linear temperature gradient (0.5°C/30 s) using excitation and emission windows set at 560−590 and 610−650 nm, respectively. Melting temperatures (T_m_) were calculated by fitting the sigmoidal melt curve to the Boltzmann equation as described using GraphPad Prism, with R^2^ values of >0.99^40^.

### NMR experiments

EPAC1_h_ 149-318 was expressed and purified using previously documented protocols^24^,^25^,^26^,^27^ and dialyzed into NMR buffer (50 mM Tris, 50 mM NaCl, 2 mM EDTA, 2 mM EGTA, 1 mM DTT and 0.02% NaN_3_, pH 7.6). NMR experiments were performed with 100 μM EPAC1_h_ 149-318 in NMR buffer with 5% D_2_O and 50 μM of the referencing standard ^15^N-acetyl-glycine. Spectra were acquired at 306 K on a Bruker Avance 700 MHz spectrometer equipped with a 5 mm TCI cryoprobe. Gradient- and sensitivity- enhanced {^15^N, ^1^H} heteronuclear single quantum coherence (HSQC) spectra with 128 (t1) and 1024 (t2) complex points and spectral widths of 31.82 and 14.06 ppm for the ^15^N and ^1^H dimensions, respectively, were recorded with 8 scans and a recycle delay of 1 s. For 25 μM EPAC samples, spectra were acquired with 256 scans. All spectra were processed with NMRPipe^41^ and analyzed in Sparky^42^.

## Author Contributions

Y.Z., H.C., G.M., J.Z. and X.C. participated in research design. Y.Z., H.C., S.B., F.M. and N.Y. conducted experiments. Y.Z., H.C., S.B., F.M., G.M., J.Z. and X.C. performed data analysis. X.C., Y.Z., S.B., G.M. and J.Z. wrote or contributed to the writing and revision of the manuscript. All authors reviewed the manuscript.

## Supplementary Material

Supplementary Information Supplementary Figure

## Figures and Tables

**Table 1:** Potency of ESI-09 analogs in competing with 8-NBD-cAMP binding to EPAC2

Compound	IC_50_ (µM)
cAMP	32.0 ± 5.9
ESI-09	9.0 ± 1.2
HJC0693	28.3 ± 4.0
HJC0694	26.5 ± 4.1
HJC0695	59.8 ± 9.4
HJC0696	7.3 ± 1.2
HJC0726	1.9 ± 0.3
HJC0750	35.6 ± 7.8
HJC0777	3.0 ± 0.6

**Table 2:** Solubility of ESI-09 and HJC0726 in water and ethanol

	ESI-09	HJC0726
**MW**	330.769	365.214
**Solubility in water**	5.95 ± 0.83 µg/mL (18.0 ± 2.5 µM)	45.4 ± 1.4 µg/mL (124.3 ± 3.8 µM)
**Solubility in ethanol**	14.98 ± 0.39 mg/mL (45.3 ± 1.2 mM)	6.8 ± 0.86 mg/mL (18.6 ± 2.3 mM)
**cLog P**	4.54	5.15
