# Supplementary material for: Biochemical and Pharmacological Characterizations of ESI-09 Based EPAC Inhibitors: Defining the ESI-09 “Therapeutic Window”
Source: Sci Rep. 2015 Mar 20;5:9344. doi: 10.1038/srep09344 (PMC4366844; doi:10.1038/srep09344)
Supplement: Supplementary Information — Supplementary Figure [file srep09344-s1.pdf]

Biochemical and Pharmacological Characterizations of ESI-09 Based EPAC Inhibitors: Defining the ESI-09 "Therapeutic Window"

Yingmin Zhu[1], Haijun Chen[2,¶], Stephen Boulton[3], Fang Mei[1], Na Ye[2], Giuseppe Melacini[3,4], Jia Zhou[2,*], and Xiaodong Cheng[1,*]

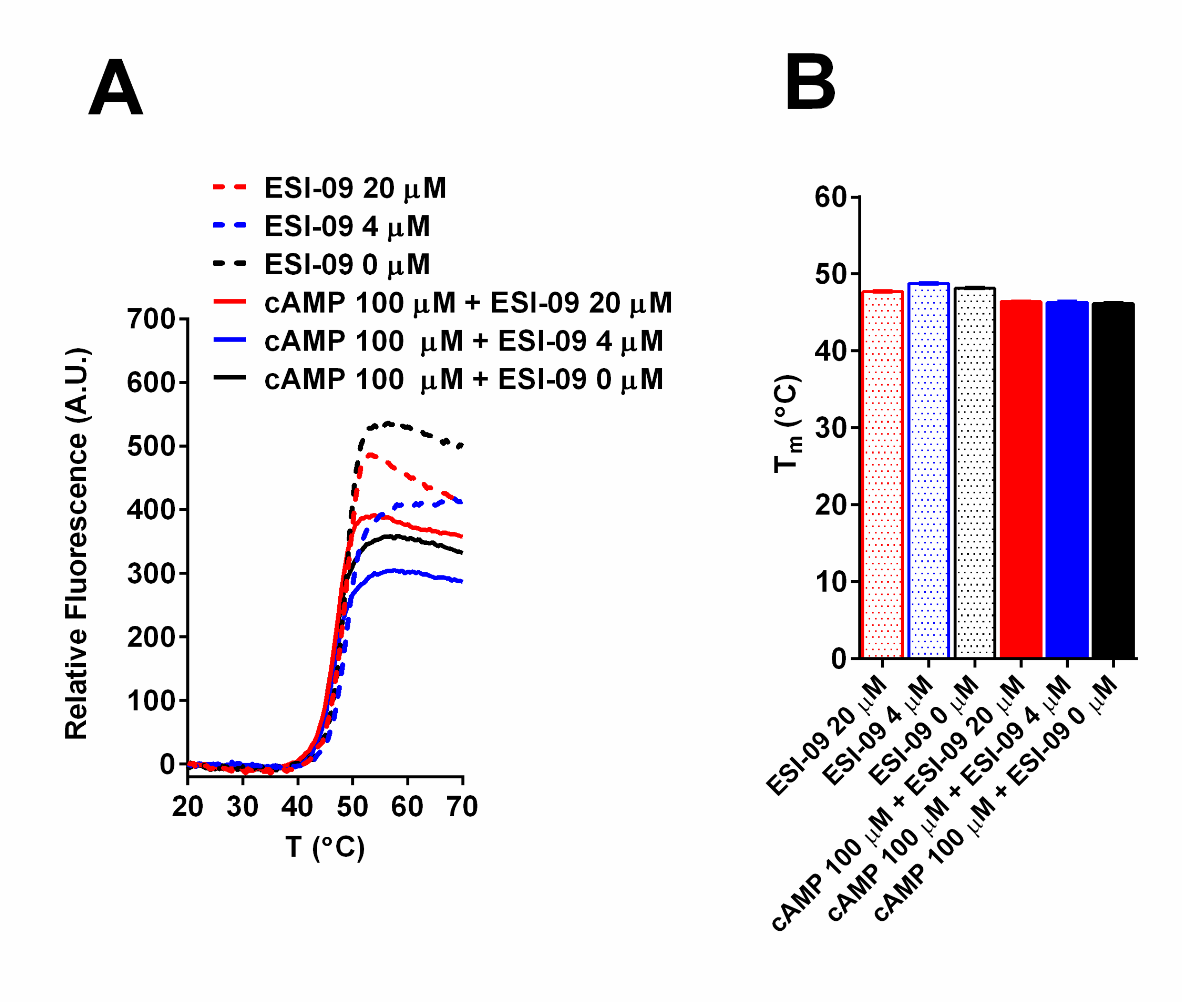

Figure S1. Effect of ESI-09 on thermal denaturation of EPAC2 in the presence or absence of 100 μM cAMP. (A) Thermal-induced protein denaturation of EPAC2 under various ESI-09 concentrations in the presence or absence of 100 μM cAMP. (B) Thermal melting temperature (T_m) of EPAC2 as a function of ESI-09 concentration in the presence or absence of 100 μM cAMP.

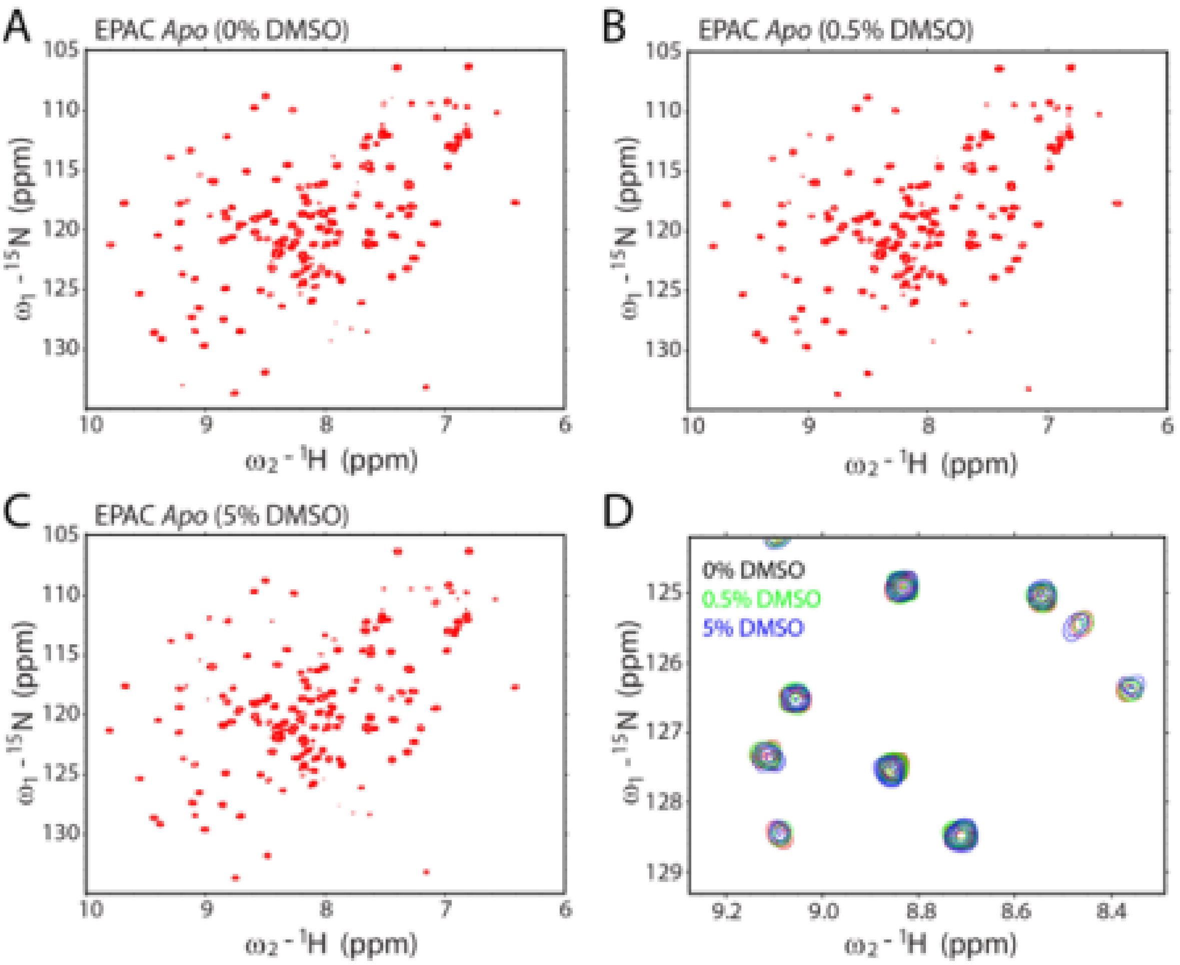

Figure S2: DMSO does not cause broadening of *apo* EPAC (149-318) resonances. {15N, 1H}-HSQC spectra for *apo* EPAC in the absence (A) and presence of 0.5% (B) and 5% (C) DMSO. (D) Representative section in the overlay of HSQC spectra from (A), (B) and (C). The presence of 0.5% DMSO results in negligible chemical shift changes for *apo* EPAC while 5% DMSO causes some modest chemical shifts, Regardless, at these DMSO concentrations, which correspond to the amounts used in Figure 5, there is no significant broadening of EPAC peaks as seen for the samples with ESI-09.
